# The 4^th^ NextGen therapies of SJIA and MAS, part 4: it is time for IL-18 based trials in systemic juvenile idiopathic arthritis?

**DOI:** 10.1186/s12969-023-00867-y

**Published:** 2024-01-05

**Authors:** Scott W. Canna, Fabrizio De Benedetti

**Affiliations:** 1 Rheumatology & Immune Dysregulation, The Children’s Hospital of Philadelphia, Philadelphia, PA USA; 2 Division of Rheumatology, Ospedale Pediatrico Bambino Gesù, Rome, Italy

**Keywords:** IL-18, IL-18 blocker, Refractory SJIA, Biomarker driven trials, IL-18ophathies, Inflammasopathies

## Abstract

Since IL-18 has recently emerged as a biomarker associated with refractory disease course in SJIA, the focus of the discussion was the feasibility of the biomarker-driven drug development to SJIA. Overall, there was broad agreement on the conclusion that IL-18 is a uniquely specific biomarker for many of the subsets of SJIA most in need of new therapies, and it may define a class of diseases mediated by IL-18 excess. The consensus was that leveraging IL-18 remains our most promising “lead” for use in refractory SJIA as it may mechanistically explain the disease pathophysiology and lead to more targeted therapies.

## Introduction

A biomarker is a “defined characteristic that is measured as an indicator of normal biological processes, pathogenic processes, or responses to an exposure or intervention, including therapeutic interventions” according to the definition provided by FDA [1]. Biomarkers have always been a fundamental aspect of medical practice and drug development, but are not direct measures of how a person feels, functions, or survives. The Food and Drug Administration—National Institutes of Health (FDA-NIH) Biomarker Working Group defines seven biomarker categories: susceptibility/risk, diagnostic, monitoring, prognostic, predictive, pharmacodynamic/response, and safety [1]. Examples include molecules (e.g. serum protein levels or DNA sequences), histologic or radiographic findings, or physiologic characteristics (e.g. fever or hypertension).

Interleukin-18 (IL-18) is an inflammatory cytokine produced by both myeloid cells (e.g. macrophages) and barrier tissue epithelial cells of the skin, intestines, and lungs. It is produced in an inactive, cytosol-bound pro-form that canonically requires inflammasome formation to be activated and secreted [2]. Outside the cell of origin, cleaved IL-18 is usually prevented from signaling by an abundant endogenous high-affinity inhibitor, IL-18 binding protein (IL-18BP) [3]. The IL-18 receptor is most prominently expressed by Natural Killer (NK) cells and activated/memory T-cells. IL-18 signaling is similar to IL-1 and most Toll-like Receptors (TLRs), but in isolation it has minimal effects compared with its ability to amplify the effects of other cytokines like IL-12. In so doing, IL-18 is perhaps best known for promoting production of Interferon-gamma (IFN-ɣ) by NK and T-cells.

Total IL-18 is readily measured in serum or plasma and normal levels (in most assays) are under about 500 pg/mL. Elevated total IL-18 levels have been observed in a host of infectious, oncologic, and rheumatologic diseases typically at levels of no more than about 10,000 pg/mL [4]. Extremely high total IL-18 was first observed in Adult-Onset Still’s Disease (AOSD) in 2001 [5], and soon thereafter in Systemic Juvenile Idiopathic Arthritis (SJIA) where the highest levels were observed in patients with Macrophage Activation Syndrome (MAS) [6]. Since that time, many groups have independently confirmed these observations (recently meta-analyzed by Krei et al. [7] and further reinforced the remarkable association of increased levels of IL-18 for SJIA and highly-similar diseases at risk for MAS [4, 7–9].

Many biomarkers also indicate clues to disease pathogenesis, providing evidence for therapeutic targeting. Indeed, evidence that IL-18 may be more than a biomarker comes from several sources. Genetic, functional, and (more recently) therapeutic evidence places IFN-ɣ centrally in the physiology of several forms of hemophagocytic lymphohistiocytosis (HLH) [9–14]. MAS is classified as a form of secondary HLH and shares elevation of biomarkers of IFN-ɣ activity (like CXCL9) with primary HLH [4, 9, 15], consistent with the role of IL-18 as a potent amplifier of IFN- ɣ production. Indeed, very high IL-18 levels may be predictive of the MAS-prone subset of SJIA [16]. The very high levels of total IL-18 in SJIA and AOSD are also associated with detectable “free” IL-18 (not bound by IL-18BP) [17], and both very high total IL-18 and detectable free IL-18 seem unique to MAS compared to other forms of HLH [4]. More recently, several independent groups have identified monogenic autoinflammatory diseases (AIDs) with recurrent MAS and chronic elevation of total IL-18 [18–22]. The unrelenting chronicity of dramatically elevated total IL-18 in these disorders suggests that IL-18 acts upstream of their inflammatory manifestations [23]. IL-18 has been available for clinical testing for several years and is increasingly part of the routine management of suspected SJIA, AOSD, or monogenic susceptibility to MAS [24].

### Why do we need IL-18 driven trials?

Establishing robust evidence of drug safety and efficacy in rare diseases is a major challenge for drug developers and a focus for regulatory agencies. Several sessions in this meeting have drawn attention to the imperfections inherent in our current SJIA diagnostic criteria [see Part I]. Diseases with heterogeneous and/or disparate clinical features, but a shared pathophysiology indicated by a specific biomarker, may be successfully “lumped” for the purpose of approval of a drug. Given this conference’s focus on refractory SJIA, it is often the most MAS-prone SJIA patients who lack arthritis and are often precluded from inclusion in SJIA trials and registries. These are precisely the patients for whom total IL-18 elevation is remarkable, specific, consistent, and often independent of other inflammatory biomarkers [16, 25, 26]. There are currently IL-18 blocking compounds and drugs that targets related or downstream pathways (e.g. IFN-ɣ blockers), that may benefit from studying diseases grouped together more by pathophysiology rather than by clinical features and/or nosographic classification.

## Summary of presentations

### The case for, and structure of, IL-18 driven trials

The initial presentation by Dr. Scott Canna began with a brief introduction to the structure of biomarker driven trials in oncology. The biomarkers in such trials included the expression of certain proteins (e.g. HER2) or the presence of specific genetic alterations identified in tumor cells [27] (Table 1). Review of the organization, outcomes, and impact of these studies identified a few key concepts. Basket trial designs in oncology have been organized in two ways: 1) biomarker-driven indications wherein a number of different tumor types were treated and analyzed together and resulted in a “tumor-agnostic” indication; or 2) biomarker-directed studies wherein patients with a specific biomarker were enrolled in the same protocol but stratified by tumor type and analyzed separately. Regardless of design, the biologic plausibility, accuracy, and prevalence of the biomarker were critical in enabling the study to proceed. Some biomarkers were developed in parallel as companion diagnostics under FDA review.

**Table 1:** Examples of successful biomarker-driven, tumor agnostic oncology trials

**Broad programs**				
NCI-MATCH	Multi-arm; assigned treatment based on specific genetic changes			NCT02465060
**Specific trials resulting in drug approvals**				
**Trial(s)**	**Design**	**Drug**	**Biomarker**	**Outcome**
KEYNOTE-158 (NCT02628067)	basket	pembrolizumab	Genetic (MSI-H, dMMR, TMB-H)^a^	FDA approval 6/2020
LOXO-TRK-14001 (NCT02122913) SCOUT (NCT02637687) NAVIGATE (NCT02576431)	basket	larotrectinib	Genetic (NTRK1/2/3 alterations)	FDA approval 11/2018
STRTRK-2 (NCT02568267)	Basket	entrectinib	Genetic (NTRK, ROS1, or ALK fusions)	FDA approval 8/2019

^a^*MSI-H* Microsatellite instability high, *dMMR* Mismatch repair deficient, *TMB-H* Tumor mutational burden high

The presentation then pivoted to a review of the many existing studies (summarized in the introduction) suggesting that IL-18 fulfilled the characteristics of biologic plausibility, accuracy, and prevalence, and could be used to group some monogenic and syndromic AIDs into a discernible diagnostic category dubbed “IL-18opathies” (Table 2). A level of 20,000 pg/mL appeared to reliably differentiate IL-18opathies from nearly all other diagnostic mimics across a range of published studies. Discussion during the session suggested a higher cutoff of 30,000–40,000 pg/mL would improve specificity but was likely unnecessary, as the only patients with levels > 20,000 pg/mL who carried “non-IL-18opathy” diagnoses would have otherwise been easily excluded, on clinical grounds, as having intercurrent severe infection or hematologic malignancy. Somewhat complicating matters are patients bearing pathogenic mutations in *PSTPIP1* who were recently shown to have chronic elevation of total IL-18, detectable free IL-18: However their autoinflammatory clinical phenotype is characterized by pyogenic arthritis and neutrophilic dermatoses, but not by risk for or features suggestive of MAS, and can be easily differentiated clinically [28].

**Table 2:** The proposed “IL-18opathies”

**Disease**	**Gene**	**IL-18 biomarker**	**Evidence of response to IL-18 blockade**
AOSD	n/a	[5, 17]	Phase II [29]
Systemic JIA	n/a	[4, 6–9, 16, 24, 26]	-
SJIA-MAS	n/a	“	-
Refractory SJIA	n/a	“	-
SJIA-LD	n/a	[26, 30–32]	Case report [33]
NLRC4-MAS/AIFEC	*NLRC4*	[18, 19, 34–36]	Case report [34]
XLP2	*XIAP*	[4, 20]	-
NOCARH	*CDC42*	[21, 22]	-
PAPA/PAID	*PSTPIP1*	[28, 37]	-

*AOSD* Adult-Onset Still Disease, *SJIA-LD* SJIA associated lung disease, *AIFEC* Autoinflammation with infantile enterocolitis, *XLP* X-linked lymphoproliferative disease, *NOCARH* Neonatal-onset cytopenia, autoinflammation, rash, hemophagocytosis, *PAPA* Pyogenic arthritis, pyoderma gangrenosum, acne syndrome, *PAID PSTPIP1*-associated inflammatory disease, *CR* Case report

Finally, the various risks and benefits of specific trial designs that would benefit from the use of IL-18 as a biomarker were discussed. Notably, there is precedent for basket trial designs within AIDs, and one such study resulted in the approval of canakinumab for Familial Mediterranean Fever (FMF), Mevalonate Kinase Deficiency (MKD, a.k.a. Hyper-IgD Syndrome), and TNF-receptor Associated Periodic Syndrome (TRAPS) [38]. Notably, this was a placebo-controlled, stratified design wherein all three subcategories underwent similar treatment and data collection but were analyzed separately. Enrollment of ≥ 22 patients per group ( ≥ 46 per diagnosis) enabled approval for three separate indications.

Potential trial designs discussed included single diagnosis, stratified basket, and diagnosis-agnostic. There are currently four ongoing clinical trials of IL-18 blocking medications in relevant rheumatic diseases (Table 3). Discussion suggested that IL-18 was likely best used as an inclusion criterion to improve diagnostic specificity. It may augment clinical findings to improve specificity, enabling enrollment of patients desperately in need of study (like refractory SJIA, including SJIA-LD) who may not have the arthritis necessary to meet even more current SJIA diagnostic criteria [39]. It may also help decrease heterogeneity by excluding SJIA/AOSD patients with lower IL-18 levels who may have a different pathoetiology and follow a different course. With several drugs approved for SJIA, the discussants noted that the more rare disease subsets were those in greater need to study. However, trials seeking an indication for refractory SJIA, SJIA-LD, or monogenic IL-18opathies, as well as stratified basket designs including these, will suffer from the same enrollment and analysis challenges. Conversely, a “diagnosis-naïve” autoinflammatory IL-18opathy basket design may suffer from irreconcilable heterogeneity.

**Table 3:** Ongoing trials of IL-18 blocking therapies

**NCT ID**	**Indication**	**Drug**	**Design**	**Inclusion/Exclusion**	**Primary Endpoints**
NCT02398435	AOSD	IL-18BP	OL –> DE	Yamaguchi [40]	AEs, change from baseline
NCT03113760	NLRC4-GOF & XIAP	IL-18BP	OL –> RW	gene, CRP or Ferritin, mAIDAI	Time to flare
NCT04641442	NLRC4-GOF	Anti-IL-1β/IL-18 bi- specific	OL –> RW	gene, IL-18, CRP or Ferritin, PGA	Time to flare
NCT04752371	AOSD	Anti-IL-18 monoclonal	OL –> DE	Yamaguchi [40]	AEs, change from baseline

*AOSD* Adult-onset Still disease, *GOF* Gain-of-function, *OL* Open-label, *DE* Dose escalation, *RW* Randomized withdrawal, *mAIDAI* Modied autoinflammatory disease activity index [41], *PGA* Physician’s global assessment, *AEs* Adverse events

### Combined IL-1β/IL-18 blockade in a patient with refractory SJIA-LD

Subsequently, Dr. Edward Behrens presented the case of an SJIA-LD patient whose lung disease appeared progressive and refractory to multiple medications. The patient was “classic” for the SJIA-LD phenotype of early-onset MAS-predominant SJIA, inflammatory clubbing, and histology proven Pulmonary Alveolar Proteinosis/Endogenous Lipoid Pneumonia. The patient’s MAS had been well-controlled on glucocorticoids and tofacitinib, but lung symptoms were disabling and objective measures of lung function (e.g. 6-min walk) were markedly affected. His serum and bronchoalveolar lavage fluid (BAL) IL-18 levels were quite elevated. After more than 6 months of treatment with an experimental IL-1β/IL-18 bi-specific neutralizing antibody obtained via extended-use authorization, the patient’s subjective improvement was substantial. Six minute walk distance and pulse oximetry were improved. Formal pulmonary function testing and chest CT were stable. He was able to wean from both glucocorticoids and tofacitinib. BAL cellularity decreased significantly. He remains on anti-IL-1β/IL-18 monotherapy with no noted infusion reactions, infections, or other side effects. Dr. Behrens noted that these data are being compiled into a case report.

### Bringing biomarker-driven drug development to SJIA

Finally, Dr. Christopher Leptak presented his perspective after > 15 years’ experience in the FDA Center for Drug Evaluation and Research (CDER) where he worked on tumor-agnostic, biomarker-driven drug trials in oncology. He noted that biomarker-driven trials should not be seen as unique to oncology drug development, and the regulatory agency is prepared to expand this paradigm. He reiterated some of the ways biomarkers might be used in clinical trials: not just for diagnostics but for stratification, monitoring, safety/tolerability. Specifically in SJIA, IL-18 may be useful as part of inclusion criteria, but could also be used within a broader study’s secondary endpoints to help stratify likelihood of response. Notably, biomarkers are often used as surrogates for treatment response, but audience members felt that the response of IL-18 to successful treatment was variable both within and between IL-18opathies, and, therefore, this possibility was discarded He noted important facets of the biomarker test itself, including testing availability, validity, stability, etc. If a biomarker will be used as part of the inclusion criteria for a registration study, it is not clear whether it needs to be developed for FDA-approval as a companion diagnostic or whether centralized collection at a CLIA-certified reference lab would be adequate. Finally, he endorsed setting up an FDA Critical Path Innovation Meeting (CPIM), which would allow investigators, patients/families, and potential industry sponsors to discuss more specific details with regulators.

## Discussions

Dr. Leptak then helped moderate a wide-ranging discussion including investigators, patients/families, and FDA representatives. The challenges of performing a “diagnosis-agnostic” autoinflammatory IL-18opathy trial were discussed. Among these challenges would be whether the heterogeneity of such a group would be acceptable to clinicians/investigators, and how such heterogeneity might affect the breadth and utility of outcome measures. However, others noted that, because the inclusion criteria for ongoing trials of IL-18 blocking therapies are so focused on monogenic diseases, they too will suffer from the increasingly-recognized heterogeneity of such patients. Additionally, the primary laboratory and clinical outcomes of these studies are quite generic (Table 3).

Finally, the discussion centered on the proper role of regulatory bodies like the FDA in these discussions. The FDA perspective, it is rightfully the task of the SJIA community (not the FDA) to decide what are the most appropriate ways to define the extent to which IL-18 would be acceptable as part of a treatment indication. He questioned whether a single, central testing site would be adequate for the needs of the trial and subsequently whether restricted test availability would necessarily restrict drug access. Additionally, the comparison to tumor-agnostic indications may not translate sufficiently to complex rheumatic diseases wherein the induction and effects of IL-18 may differ by disease. Finally, the study design remains a highly complex, open question. It was noted that two ongoing registration studies with IL-18 blockers both (perhaps reluctantly) came to a design that began with open-label (OL) treatment followed by randomized withdrawal of patients felt to “respond” in the OL (Table 3). The pros and cons of the randomized withdrawal design were discussed. The role of natural history data collection to supplement or in lieu of control groups was only briefly discussed as it was the subject of a later session.

## Summary and future directions

Overall, there was broad agreement on the conclusion that IL-18 is a uniquely specific biomarker for many of the subsets of SJIA most in need of new therapies, and it may define a class of diseases mediated by IL-18 excess. There was also broad agreement in the necessity for a CPIM meeting that included representatives of the parties involved in this session and (critically) interested industry representatives. Questions remain about 1) whether its current state of development is adequate to the task of being an essential part of a registration study, 2) how best to include it into possible trials in SJIA/IL-18opathy patients, and 3) how best to match the design of such studies with the competing interests of prospective patients and regulatory bodies. Nevertheless, despite these questions that are actively being addressed, leveraging IL-18 remains our most promising “lead” for use in mechanistically redefining these diseases and potentially disrupting a critical pathogenic node with targeted therapies to the benefit of patients who are in dramatic needs.

## Data Availability

All the data discussed during the meeting have now been published and appropriately referenced at the end of the manuscript.

## Abbreviations

AOSD: Adult-Onset Still’s Disease
TRAPS: Associated Periodic Syndrome
AIDs: Autoinflammatory diseases
BAL: Bronchoalveolar Lavage
CT: Computerized tomography
DNA: Deoxyribonucleic Acid
FMF: Familial Mediterranean Fever
CDER: FDA Center for Drug Evaluation and Research
CPIM: FDA Critical Path Innovation Meeting
HLH: Hemophagocytic lymphohistiocytosis
IFN-ɣ: Interferon-gamma
IL: Interleukin
LD: Lung disease
MAS: Macrophage Activation Syndrome
MKD: Mevalonate Kinase Deficiency
NIH: National Institutes of Health
NK: Natural Killer
OL: Open-label
SJIA: Systemic Juvenile Idiopathic Arthritis
TLRs: Toll-like Receptors
FDA: U.S. Food and Drug Administration
